# *“Learning by Design”*: What Sports Coaches can Learn from Video Game Designs

**DOI:** 10.1186/s40798-021-00329-3

**Published:** 2021-05-26

**Authors:** Sam Robertson, Carl T. Woods

**Affiliations:** Institute for Health and Sport, Victoria University, Melbourne, Australia

**Keywords:** Practice, Interdisciplinary, Skill, Training

## Abstract

There have been multiple calls made in the sport science literature for the promotion of interdisciplinarity to progress some of sports’ most prevailing challenges. Designing practice environments that support learning represents one such challenge, particularly given contemporary perspectives of skill acquisition and motor learning calls for coaches to realign their role—progressing toward the *designers* of practice tasks that promote athlete-environment interactions. In doing so, performers learn through exploration, deepening a relationship with their performance environment as they solve problems based on changing and interacting constraints. This paper illustrates an interdisciplinary approach to the area of learning through sport practice by adapting established principles embedded in video game designs. Specifically, 13 principles common to *good* video game designs are described, with practical examples of each provided across different sports. Fundamentally, this paper aims to offer sports practitioners with an overview and application of key principles that could support *learning by design*. Beyond this, the ideas presented here should further illustrate the value of interdisciplinarity in sports research and practice.

## Key Points

- Using an interdisciplinary approach, this paper shows how principles embedded in good video game designs can be applied to sport to support athlete learning and performance.
- Each principle is accompanied with a unique sporting example—exemplifying what they may ‘look’ like for sports coaches seeking to bring them to life in practice.
- This paper should be seen to promote interdisciplinarity within sport science—drawing on other disciplines to help progress disciplinary challenges.

## Introduction

Over the past three decades, multiple calls have been made in the sports literature for the promotion of interdisciplinarity in order to help progress some of sports’ most prevailing challenges (see [1–6]). Designing appropriate learning environments for performers across the participation pathway represents one such challenge for many individual and team sports. Part of the difficulty in this task relates to the complexity of sporting environments [7], which require performers to develop knowledge of interacting constraints that evolve along different timescales of learning and performance [8]. Thus, the development of sporting skill is a process that can take many years invested across a diverse set of experiences and landscapes [9].

Despite this, traditional perspectives on learning in sport have tended to advocate a rather coach-centred approach, whereby a coach relies on giving direct instruction and constant, prescriptive feedback with the intent of shaping behaviour around a technical behavioural model [10, 11]. This pedagogical approach not only implies that the coach is all-knowing, but also that it is a core function of their role to constantly convey declarative knowledge to the performer about how to solve performance-related problems in a ‘global’ or ‘top-down’ way. The inherent reductionism and linearity of such approaches has drawn scrutiny in recent years [12–15].

In light of this scrutiny, contemporary perspectives on learning and performance in sport have sought to embrace inter- and even transdisciplinarity; blending disciplinary ideas, methodologies and concepts to search for new ways to progress beyond traditional dependencies [16]. For example, there are growing calls for coaches to view themselves as *learning designers* who place the performer-environment interaction at the core of their design [17–19]. These ideas, grounded in an ecological dynamics rationale [17, 20, 21], implicate how learning and practice environments can be understood, progressing from more artificial means of learning by rote, to the *design* of information-rich landscapes that promote search, discovery and adaptability in the solving of novel performance problems [17]. This provides performers with opportunities to learn through exploration [22], progressively understanding how to solve problems by actively self-regulating their perceptions, cognitions, emotions and actions toward the achievement of a task goal [17]. Simply, what is ‘acquired’ during skill acquisition is a deep and evolving relationship between a performer and the constraints of their environment [12, 17].

This paper aims to show how principles embedded into good video game designs can be applied to sport to assist the development of learning and performance when conceptualised through an ecological dynamics rationale. In doing so, it promotes interdisciplinarity, demonstrating how established principles of learning embedded from one area can be transported to support another, which encourages practitioners to conceptualise themselves through a *learning designer* lens.

## What Sport can Learn from Video Game Designs

The intentions of this paper were inspired by James Paul Gee [23], who proposed that the intricacies of good video game designs exemplified established principles of human learning. His work proposed 13 principles that video game designers often harness to engage and empower the players navigating their games. Importantly, although situated within video games, he argued that these principles could and should be applied to learning in other domains. For example, applied through a Games-Based Approach, Price and Pill [24] developed a coaching pedagogy based on Gee’s principles, and sought to apply them. Although this pedagogy supported the development of empowerment, problem-solving and understanding, the authors noted “…it was not clear in this study how *all* elements of Gee’s good game design can be meaningfully translated from the digital to the ‘physical’ sport coaching practice context” [p. 257, our emphasis]. In this paper, we therefore look to extend these ideas through an ecological dynamics rationale, exemplifying what each of the 13 principles could ‘look’ like for sports coaches across a variety of different sporting contexts. Specifically, the below adaptation to sport presents these principles in a user-friendly format, in order to simply assist coaches and practitioners in the design of effective learning activities in their sport. As was done in the initial article by Gee [23], these principles are framed beneath three themes—Empowered Learners, Problem Solving, and Understanding. Accompanying each principle are, thus, examples of how it could be applied to sport practice.

## Empowered Learners

### Principle 1: Co-design

A goal of any good learning design is to develop intelligent performers [25]. Rationalised through ecological dynamics, an intelligent performer is an adaptive, engaged and motivated individual who learns quickly and relies on perceptions, cognitions, emotions, and actions to function effectively in a specific environment [25, 26]. As proposed by Gee [23], good video game designs support this, as players feel like their actions and decisions are indeed their own, not just those of the game’s designer that they then simply enact in practice. To foster the development of this intelligence, individuals could be included in the design of their learning activities, being invited to consider how to *co-design* features of the environment that could be utilised to promote certain behaviours. This is likely to support engagement, as the individual could feel more like an active participant who has a shared responsibility for the design of their learning activities, rather than solely a passive consumer of continued instruction provided by an educator [25].

#### Principle in Sport

A coach of an elite basketball team could work with a point guard to *co-design* a practice task intended to challenge an offense. Specifically, the coach could ask the point guard which defensive structures they feel challenges both them and their teammates’ capability to keep the ball in motion and work it into a position that enables a shot. Once these structures have been collaboratively identified, the coach and player could co-design them into a practice task. The coach could then observe how the point guard (and team) explore ways of overcoming the defence during the task, while additionally asking them after the task (ex situ) to describe how ‘game-like’ and challenging it was, and what features could be changed or manipulated. This questioning could also be used by the coach in situ (during performance) to encourage the player(s) to explore the various regions of the performance environment that may have been co-designed in. Importantly, the ‘answers’ to such in situ questions need not be verbalised by players, but rather ‘actioned’ in how they search for, discover and exploit various opportunities for interaction with their performance environment [14, 25]. Combined, this information could then be used as a basis to inform future designs intended to challenge the team’s offense.

### Principle 2: Customize

Individuals are unlikely to become self-regulating performers if they do not make decisions about how, where and when their learning will take place. From an ecological dynamics rationale, ‘self-regulating’ refers to the regulation of perceptions, cognitions, emotions and actions, developed through carefully designed practice tasks [17]. Thus, to support the development of such active self-regulation, at times, performers could be encouraged to *customize* the delivery of information offered by the educator to match their perceptions of what they may or may not understand. In doing so, the performer could be encouraged to explore new and innovative ways of learning that the educator was yet to consider.

#### Principle in Sport

The facilitation of athlete-led training sessions could provide them the freedom of designing activities they feel may improve their performance. In such sessions, the coach simply observes the athlete(s) and only offers guidance when or if asked. For example, a goal shooter in netball could be free to *customize* the difficulty of a practice task intended to improve their shooting accuracy, being encouraged to make it easier or harder based on the number of (un)successful shots taken. However, while coaches may not be actively scheduling practice in such sessions, it would be important for them to ensure that the practice task representativeness is functionally preserved through careful nudging if/when required (i.e., asking the goal shooter whether they can perform the shot from different court locations if prolonged repetition is noted).

### Principle 3: Identity

An individual’s learning can be supported through the establishment of an *identity* in their environment and among their peers [23]. An identity is likely to engage the performer with a specific aspect of their learning environment, guiding their attention toward its maintenance. As proposed by Ryan and Deci [27], this could encourage the performer to take greater ownership of and responsibility for the learning environment, situating their behaviours and learning in such a way that motivates them to bring life to, and maintain, their identity.

#### Principle in Sport

Instead of coming directly from a coach, athletes could develop their own strategic principles based on values they perceive as critical to an *identity* they choose. For example, a rugby league team could choose to identify their attacking principles of play as being ‘creative’ and ‘exciting’. This would encourage them to search their practice and competition environments for opportunities to exhibit such actions when in attack, but not constrain them to a rigid, pre-determined model of doing. As another example, a young player who identifies as a future leader may seek out opportunities within the training environment to further develop this skill set, thus also benefiting the team.

### Principle 4: Manipulation and Distribution of Knowledge

A key contention of behaviour when viewed from an ecological dynamics rationale, is that perception and action are tightly coupled [17, 20, 21], meaning that as individuals move, they continue to perceive opportunities to act and vice versa [20]. Learning, from this perspective, is therefore an exploratory process, where individuals progressively learn to perceive various opportunities for action that change as their action capabilities change [17]. Accordingly, during performance preparation, individuals should be exposed to numerous opportunities in which to *manipulate and distribute their knowledge* of a variety of different perspectives or performance landscapes [28]. This will likely ‘tune-in’ their perceptions in relation to their action capabilities, learning to become more attentive to a variety of opportunities for action when attempting to achieve a particular task goal. To help support the development of varied performance landscapes, performers could be encouraged to manipulate environmental features or distribute their knowledge across different features of the learning process in an effort to help extend and grow their action capabilities [29].

#### Principle in Sport

Athletes could be encouraged to take on the role of the coach. In this way, the athlete would be responsible for designing a learning activity, including its intentions and task constraints, while concurrently having to consider how to affect its design in real-time if required. This will distribute the athlete’s knowledge of the game, viewing it from the perspectives of the coach, who is likely to directly perceive different features of the game and subsequent possibilities for action from their vantage point. Another example could be a coach manipulating the roles/positions of players in team games to distribute a player’s knowledge of the game (i.e., defenders playing as forwards and vice versa).

## Problem Solving

### Principle 5: Well-ordered Problems

If anything is acquired during skill acquisition, from an ecological dynamics perspective, it is a functional and evolving relationship formed between a performer and their performance environment, learning how to adapt movements based on emergent problems encountered [12]. Therefore, problems designed into learning activities need to be closely matched or aligned to a performer’s action capabilities [30, 31]. If the problems are too complex, the performer may be unable to find a way to solve it or do so in a way that leads to negative experiences that could hinder future problem-solving opportunities.

#### Principle in Sport

An important feature of successful offensive behaviour in football orients ‘tempo control’—defined here as being attuned to opportunities that invite fast or slow ball movement based on a range of interacting constraints, like an opponent’s defence and time of game. Through the use of *well-ordered problems*, a coach could help players recognise when these opportunities emerge during game-play. For example, a coach could start a task by informing an offensive team that the defence will be either two players up or down, allowing them the opportunity to strategize potential solutions (i.e., determining if they will likely have opportunities to move the ball fast or slow based on a known problem). Once a certain level of success is observed in these more predictable problems, the coach could randomly add or remove defenders without informing the offensive team during the practice task. This creates more challenging and dynamic problems for the offensive team, having to learn to attune to the opportunities to increase or decrease ball movement speed ‘in-game’ rather than discussing possible solutions to known problems ‘out of the game’. This also emphasises the role of the coach as one very much aligned to that of a *designer*—in that problems are progressively incorporated into the practice task without pre-defined solutions, encouraging players to deepen their knowledge of the performance environment and its emerging (and decaying) opportunities for action.

### Principle 6: Pleasantly Frustrating

Learning is likely to occur when new and *pleasantly frustrating* problems are encountered. These are problems that are perceived to be just out of the reach of the performer’s current action capabilities, but still within their ‘regime of competence’ [23]. More directly, these problems are challenging, but doable for the individual [31]. In good video game designs, Gee [23] argued that such problems often manifest in players having to overcome a ‘boss’ or perform a particularly difficult task, which often takes multiple attempts to achieve. Thus, although such problems may lead to acute performance ‘failure’, they offer the performer progressive evidence that they are getting closer to successfully solving them [32]. This aspect is crucial to such problems, as it is this progressive evidence that guides the performers’ attention toward features of the problem that could be exploited to solve it in future attempts—thereby engaging and likely motivating them.

#### Principle in Sport

A coach could challenge an aerial gymnast to incorporate a movement within a routine that is slightly beyond their current action capabilities. Importantly, while the athlete is learning how to solve this problem, the coach should facilitate a safe but uncertain environment, where the athlete can ‘fail’ without placing themselves at heightened risks of judgement or injury. The coach could then ask the gymnast how pleasantly frustrating the problem is, enabling them to manipulate features of the problem to surf its challenge point [31], thereby keeping it within the ‘Goldilocks zone’ (i.e., not too hard, but not too easy).

### Principle 7: Cycles of Expertise

A movement is typically adapted from more stable behavioural states, which are developed through *repeated cycles of expertise*. Thus, educators could facilitate an environment that progressively challenges stable movements, which encourages a performer to adapt them in novel ways [17]. Although this will likely incur some ‘failure’, it will minimise performance plateauing, and encourage the performer to continually search for adaptive movement solutions.

#### Principle in Sport

The coach of a junior tennis player could manipulate the quality of the player’s opponent. For example, if the junior player is rarely challenged to adapt their movements against opponents at their current playing level, the coach could incorporate practice sessions against older, more experienced players. This process of playing against junior and senior players could be continually cycled, encouraging the junior player to engage in differing levels of movement adaptation based on their opponent’s level of expertise.

### Principle 8: Information “On-Demand” and “Just in Time”

It is well established that the timing and frequency of information or feedback given to individuals impacts the learning process [33]. Specifically, information and feedback should be given to performers *on demand* (i.e., when an individual feels they need it) and *just in time* (i.e., when they can put it to use) [34]. These feedback frequency and timing strategies can engage and motivate the performer, reducing their reliance or dependency on an educator when solving a problem, if used correctly [35].

#### Principle in Sport

The coach of a snowboarder could design the problem of learning a new trick to increase the athletes’ point scoring capacity during competition. Prior to attempting the trick in a practice task, the coach could provide initial information to the athlete about how they may want to perform it. However, during the practice task, the coach would encourage the athlete to explore ways of performing the trick, only providing feedback when the athlete asks (e.g. *on demand*), and in such a way that they can use the information to guide their attention toward things in future attempts (e.g. provided *just in time*). From this perspective, the coach needs to ensure they provide enough information at the start of the task so the athlete can progressively learn the trick (e.g. ‘showing them where to look’), but not so much that the athlete develops an overreliance on their feedback (e.g. ‘telling them what to see’) [36], hindering their exploratory tendencies during the learning process.

### Principle 9**:** Fish Tanks

*Fish tanks* represent a simplified marine ecosystem, where critical interactions between organisms and the environment are present and easily identified by an observer. Applied to learning designs, this metaphor illustrates the importance of creating simplified, not deconstructed, practice tasks. In good video game designs, examples of fish tanks may include preliminary levels, or tutorials, where players can explore action capabilities in a representative, but simplified, environment [23]. Specifically, critical features and their interactions are still preserved within the practice task, but they are designed in such a way that the performer can more easily identify and interact with them. More directly, these tasks represent a simplified version of a broader skill to be learned (interested readers should consult [37] which discusses the importance of task simplification for learning in sport).

#### Principle in Sport

Within sports such as rhythmic gymnastics or figure skating, athletes are often required to couple movements to music in order to create a routine. The central feature of this task is, therefore, the interaction between the athlete and the music. Adopting the fish tank principle, a coach could simplify the practice task for the athlete when they are initially learning a new routine by slowing the music’s tempo. Doing so may allow the athlete more time to couple their movements to components of the music, preserving the fundamental interaction between the athlete and the environment in a more manageable way. As the athlete tightens the movement-music coupling, the coach could progressively vary the music’s tempo, while also adding more features of the environment that could impact the routine, such as awarding or reducing points for movements and/or adding spectators.

### Principle 10**:** Sandboxes

*Sandboxes* metaphorically highlight the importance of individuals being placed in authentic (i.e., ‘representative’ from an ecological dynamics perspective [17]) environments where they feel safe to explore, create and fail without negativity or reproof. From a learning point of view, such an environment promotes safe, but still uncertain self-guided exploration and discovery. Moreover, given the authenticity of the environment, the performer should feel free to deepen their knowledge of their action capabilities embedded within a representative and meaningful context [38].

#### Principle in Sport

Given increasing technological advancements in sport, virtual reality environments are becoming a more accessible practice tool for coaches and athletes. Such environments have the advantage of situating the athlete within an authentic environment while mitigating risks of injury associated with exploratory behaviour when learning a new skill. For example, a baseball coach could consider the use of virtual reality for a young batter who is struggling to hit faster pitchers to certain field locations (interested readers could consult the work of Gray [39], which offers empirical insight to this example). Given the better is facing a (virtual) pitcher and required to play a (virtual) shot, this enables an environment that somewhat preserves the fidelity of the action but does not place the hitter at risk of injury (i.e., being physically hit by the ball). It should be noted, however, that virtual environments are complementary and should not simply replace learning by ‘doing’ in ‘real world’ sporting environments. Moreover, careful consideration should be directed toward ensuring the fidelity and functionality of perception-action coupling when designing and using such virtual environments.

### Principle 11**:** Skills as Strategies

Skills do not occur in a vacuum, but are emergent properties of the individual-environment interaction [40]. Although high levels of practice are required to learn a skill, educators need to ensure that the practice task has reference to the specific environment or context in which it would be used or performed [14]. More directly, practice tasks should be embedded [38], with the performer placed in a representatively designed environment [41, 42], encouraging them to practice *skills as strategies* toward the achievement of a task goal [23]. Thus, this principle appreciates the reciprocity of the performer and the environment, emphasising that what is progressively acquired is not an action template removed from context, but is a continually deepening and functionally adaptable relationship with a niche in performance environment [12].

#### Principle in Sport

A functional skill in soccer is ‘dribbling’ the ball—that is, maintaining ball possession while running. Considering this *skill as a strategy*, teaching young players how to dribble requires an environment that promotes its repetition, but also ensures that players understand how dribbling emerges with consideration to match context, opponents and teammates (i.e., representative practice task design [42]). Simply practicing the dribbling action removed from such context and intent (i.e., dribbling to a stationary cone and back) reduces its strategic meaningfulness for the player. Modified games present one opportunity by which this skill can be better utilised as a strategy. For instance, the coach could award points to players who are able to maintain ball possession while dribbling to avoid defenders for defined periods of time. In doing so, players would be repeatedly exposed to the problem (e.g. challenged to maintain ball possession), but in a way that sees them learning to solve it in different ways (for a detailed insight here, see the second case example in [43]).

## Understanding

### Principle 12**:** System Thinking

From an ecological dynamics perspective, athletes and sports teams are conceptualised as complex adaptive systems [17]. This means that behaviour is understood non-linearly, with changes in system properties having non-proportionate effects on how the system behaviours. Given this, practice tasks need to be contextualised for performers [17]. Moreover, such practice tasks need to promote *systems thinking* or conceptualisation by presenting the performer with the opportunity to understand how they, or the skill they are learning, fits into a broader ecology of relations [7]. For example, an individual learning to paint may need to understand how certain brush strokes and colour spectrums can be used to create an identifiable ‘bigger’ picture. Thus, practice designs need to facilitate opportunities for the learner to understand how a ‘to-be-learnt’ skill fits within, or implicates, a larger system of behaviour.

#### Principle in Sport

Many team sports require players to have position-specific skills. For example, in Australian Footbal, ‘ruckmen’ are often physically taller players who compete in an aerial contest to ‘tap’ or ‘knock’ the ball to teammates who are usually midfielders. A coach promoting *system thinking* within a developing ruckman could design a practice task that not only encourages them to practice the action of ‘tapping’ the ball, but incorporates midfielders and opponents of differing configurations to deepen their understanding of how the ball's placement following the ‘tap’ affects the likelihood of their teammates obtaining it. By situating the player within this ruckman-midfielder system, and encouraging its exploration, the player will likely deepen their knowledge of how ‘tapping’ the ball can be adaptively performed in such a way that positively implicates a broader system of behaviour (including consideration of the opposing team).

### Principle 13**:** Meaning as Action Image

For humans, analogy and metaphor can offer a powerful basis to support learning [14]. For example, when learning to set the ball in rugby league following a tackle, a coach may analogously ask the player to “set the ball in concrete” ([14], p. 132); an informational constraint that helps the player ground the *action* within a context that is of *meaning* to them. Thus, a human’s understanding of a ‘thing’ is typically shaped by their direct experiences of it, not generalisations or abstractions of it [20, 21]. Gee [23] emphasised this within the design of good video games, stating that “words and concepts have their deepest meanings when they are clearly tied to perception and action in the world” [p. 14]. Learning designs should, therefore, be situated in context and meaning to help performers explore various regions of their performance environment. The goal of the practitioner, then, is to design information rich practice tasks that situate *action in meaning*, specific to the performer.

#### Principle in Sport

A coach can guide or nudge an athlete toward the coupling of their perceptions and actions by harnessing *meaning as an action image* through carefully integrated analogy and/or metaphor [14, 17]. For example, a coach of a young cricketer learning to catch a ball while fielding at ‘slip’ may encourage them to position their hands relative to the ball when it approaches them *‘like the ball is an egg not to be cracked’*. This analogy, anchored in meaning for the young cricketer, may lead them to self-organise their interaction with the ball to catch it softly, minimising excessive reaching or grasping that could emerge with direct, prescribed, mechanical instruction. Moreover, this analogy preserves the coupling of perception and action, as the words are clearly aligned to an action (of catching an egg) for the cricketer, while being situated in context (standing in slips, waiting to catch the ball).

## Conclusion

Contemporary perspectives on learning and performance in sport have sought to embrace interdisciplinarity, integrating ideas from other disciplines to search for new ways to progress beyond the traditional ‘ways of doing’. This has led coaches to conceptualise themselves through a *designer* lens, facilitating practice tasks that promote athlete-environment interactions. This paper adapted 13 principles germane to good video game designs, showing how such principles could be applied to sport to support coaches who view themselves as learning environment *designers*. Importantly, these principles demand that skills be repetitively practiced, but done so in a way that presents the performer with the opportunity to deepen their knowledge of the interactions between the ‘to-be-learnt’ skill and the environment. Simply, they advocate a type of repetition without repetition [44], and as such, align with pedagogical approaches common to a non-linear pedagogy, such as the constraints-led approach (for further reading on non-linear pedagogy, see [14, 15]). Moreover, while discussed separately, each principle should not be viewed independently from one another, but rather as intertwined tools that coaches can draw on when designing practice tasks in sport. For example, an athlete could be encouraged to *co-design* a practice task that offers *pleasantly frustrating* problems to them and/or teammates to solve within a *fish tanked* environment.

To conclude, we highlight some common threads linking each of the principles described here. Namely, each principle promotes *self-discovery* and places the *performer at the core* of the learning design. Both threads encourage performers to problem solve, explore and create novel ways of doing through carefully (co)designed practice tasks. This ultimately contributes to the development of a self-regulating performer—that is an individual who learns to self-regulate their perceptions, emotions, cognitions and actions as they navigate the many dynamic environments sport presents.
